# The Health of London

**Published:** 1921-12

**Authors:** 


### THE HEALTH OF LONDON.

THE publication of the Annual Report of the Medical Officer of Health (Dr. Hamer) of the London County Council is always an event in the public-health year. Dr. Hamer has original views, and is not content to follow unthinkingly the "use-and-wont" theories of disease and epidemiology. His Report for 1920 is a document of supreme interest, and we therefore give below certain extracts from it. The Report itself should be read in full and carefully by all who are concerned in the prevention of disease. The volume can be obtained for the modest sum of half-a-crown from P S. King and Son, Ltd., Great Smith Street, Westminster. We propose to consider in a future issue the portions of Dr. Hamer's Report which deal with the School Medical Service of the London County Council. Dr. Hamer writes:—

The view has been consistently maintained in these reports, for the last ten years or more, that (in London at any rate) typhoid carriers, flies, milk, ice-cream, and watercress, have not been responsible for the spread of typhoid fever. Furthermore, that the part played by direct case-to-case infection cannot be in any case an important one; while the influence of preventive inoculation has been altogether negligible. With these considerations in mind it was possible, in 1914, to reach the conclusion that the effect of the war was not likely to be to increase, as some supposed, the risk of spread of the disease, but to diminish it. This expectation has been realised. Moreover, as an outcome of the experience gained in the last seven years, the vogue of the carrier hypothesis has materially lessened; the conviction having steadily gained ground that typhoid fever is for the most part water-borne and food-borne; there remains still, however, in many minds the belief that case-to-case infection is quite commonly met with, and that important influence, so far as males are concerned, has been exerted in the last few years by protective inoculation. London experience does not lend support to either hypothesis (see Report, p. 25). We are led, moreover, irresistibly to entertain the suspicion that "Even in a world-wide pandemic the problem of epidemic influenza may be largely an internal problem of each nation, a problem of social relationship, of social factors, of domestic habits and life. The materials of the conflagration come from within. As the compilers warn us, the wolf is in the fold all the time. "His ravages depend as much upon the disposition of the sheep within the fold as upon his appetite."

With the conviction that this view of the matter embodies the most important practical lesson to be learnt from the epidemiology up to date, attention has been concentrated in London upon safeguarding, wherever possible, the disposition of the sheep within the fold. It is quite clear that, whatever may be done in the future, there is no sign up to the present time that prophylactic vaccination is of any value whatever. The one plain indication is just to reduce as far as may be the danger to be apprehended from badly ventilated and overcrowded places of assembly. Thus every effort should be made to secure satisfactory mechanical means of ventilating rooms and passage-ways which are apt to be thronged by large numbers of people. Every effort, too, should be made to resist curtailment of the very modest requirements laid down in statutes and by-laws with regard to cubic and floor space and the height of rooms. It was ascertained, on the instruction of the Public Health Committee, that so far as the Tube Railways were concerned careful consideration was already being given to the problems involved. Under instructions from the Theatres and Music Halls Committee renewed examination was made of the atmosphere of theatres, etc., in which necessary works had been postponed during the war years. A deputation from the Council attended upon the Minister of Health to deprecate any weakening of the requirement in the Building Act as to the minimum height of rooms. In the existing state of knowledge it is in directions such as these that the evil consequences of influenza prevalences can best be countered. There can be no doubt that influenza has been far more widely spread than is generally realised in London since the third considerable outbreak of the present series (that of February-March 1919), but the epidemic influenza has been in many instances cloaked in the guise of the gastro-intestinal and nervous forms which influenza is apt to assume. There is reason for thinking that some of the cases, at any rate, of glandular fever and of anomalous scarlatinal sore throat, as well as much of the so-called epidemic hiccough, epidemic lethargy, poliomyelitis and cerebro-spinal fever of 1920 were in fact of influenzal origin. Fortunately the case mortality has remained quite low. Cerebro-spinal fever itself has, as is its wont, been comparatively unobtrusive during the swell of the great influenzal wave which culminated in 1918 and in the years immediately following.

It is the duty of a midwife to advise that medical help is required when inflammation of the eyes, however slight, occurs in infants. Great stress has been laid by the Council on the importance of strictly observing this rule. When a notice is received from a midwife that medical assistance has been sought on this account, the Council's medical inspector at once visits the infant to ascertain that it is receiving the medical assistance which the midwife has advised, and the medical officer of health of the borough in which the patient resides is immediately informed of the condition of the case and whether the infant is being removed to a hospital for treatment. The midwife is also visited to learn particulars as to the case, the antiseptic precautions taken beforehand, and the disinfection adopted by her after each of her

**The Health of London—(*cont.*).**

attendances. It was found in the past in some cases that when medical assistance was obtained, the medical practitioner, after examining the infant's eyes, gave directions to the parents as to the course of treatment, and left instructions for them to seek medical help again if the inflammation did not yield to treatment, and if there was no improvement in the condition of the child's eyes. So long as the midwife remained in attendance, the treatment was for the most part carried out, at all events once a day; but many of these cases had not recovered by the tenth day, the time the midwife usually ceases her attendance. It was also found impossible for the midwife personally to attend to the infant's eyes as often as was requisite—i.e., in some cases four or five times daily—and this duty was then left to the friends of the patient, who were utterly ignorant of the need for using clean rags and sterile water.

It is in cases of this description that the voluntary nursing associations have rendered most valuable assistance. Practically every street in the county is now covered by the activities of one or another of these, and arrangements exist between the boroughs and various agencies by means of which a nurse can always be obtained when wanted for the home treatment of a case of ophthalmia neonatorum. In two districts (Bermondsey and Southwark) the boroughs have their own nurse available for such cases, instead of contracting out for such services.

During the year 1,079 notices were received from midwives, stating that medical assistance had been advised on account of inflammation of the eyes of infants, and, in addition to these, thirty-nine other cases came to light in which medical help was not called by the midwife.

Of these 1,118 cases, 684 proved to be ophthalmia neonatorum; in addition 480 cases of ophthalmia neonatorum not occurring in the practice of midwives were notified, making a total for the year of 1,164 cases of ophthalmia neonatorum. The percentage occurring in the practice of midwives was thus 59 per cent. for the year.

The Council's inspectors obtained 1,483 samples from churns of milk consigned from the country to the various London railway termini, and these were submitted to the Lister Institute for bacteriological examination. The milk was sent to London from thirty-six counties. Of the 1,294 completed examinations, 76, or 5.9 per cent., yielded tubercle bacilli, as against 6.5 per cent. in 1919, 7.4 per cent. in 1918, and 10.3 per cent. in 1917. In connection with these samples the veterinary inspector visited sixty-seven farms and examined 2,737 cows. It was found that sixty-three cows—i.e., 2.3 per cent.—showed signs of tuberculosis in one or more of its forms, or were otherwise unhealthy. In the case of each cow suffering from tuberculosis the farmer undertook to have the animal slaughtered, and meanwhile not again to use the milk. In addition, the inspector revisited 326 farms and inspected 14,352 cows; all the animals examined at these revisits were found to be in a satisfactory condition with the exception of 145, which appeared to be tuberculous. In each instance the owner undertook to have the animal removed.

During the year 1920 there were four inspections of the cows in the 108 London cowsheds. In all 7,120 examinations were made. No cases of tuberculosis of the udder were detected, but in 144 cases other unhealthy conditions of the udder were found. One cow was found presenting symptoms of general tuberculosis.

Much pioneer work in the biological treatment of sewage was done by the Council's officers, and before the war important questions arising out of their experiments were being considered. A new process was then being developed which, by prolonged aeration of sewage, produced an inoffensive sludge, quite different from ordinary sewage sludge, and an effluent of excellent quality. This treatment, generally known as the "activated sludge" process, alters the physical and bio-chemical features of the suspended matters, and they become purifying agents. Successful results on a small scale were obtained in the Council's laboratories, but the outbreak of war suspended the work.

